# Our Senior Doctors

**Published:** 1895-02

**Authors:** J. Bailey Sullivan

**Affiliations:** Vorona, Pa.


### OUR SENIOR DOCTORS.

J. Bailey Sullivan, M. D., Vorona, Pa.

We see so few contributions in our medical journals from the pens of many widely-experienced practitioners of our own acquaintance—doctors whose varied and successful practice, if duly recorded in our medical literature, would serve as beacon lights for coming generations—that the question has often come to our mind, Why is this thus?

This seeming omission of professional zeal, in most cases, is, no doubt, due to want of time. As a matter of course, it cannot be expected that such physicians can afford to sit for hours writing lengthy articles, which, as a rule, are little read and less valued; but those busy doctors could easily find leisure—if inclination prompted—to pencil briefly a few lines, occasionally, regarding the *verification* of a certain drug which they found serviceable in some special case. Thus did the great fathers of Homœopathy in bygone days; thus was our materia medica gradually built up, enriched, and brought to its present state of comparative excellence; and were our distinguished seniors of to-day to do likewise they also would render the profession an enduring benefit, our medical journals would come to us laden with choice and miscellaneous bits of knowledge, and many of our local leading physicians may in the course of time acquire a national reputation, and have their names honorably chronicled in homœopathic bibliography.

